# Human *Streptococcus suis* Infections, South America, 1995–2024

**DOI:** 10.3201/eid3107.241835

**Published:** 2025-07

**Authors:** M’hensa Vincent De Paul Bakpatina-Batako, Kevin Li, Sonia Lacouture, Lucía Cipolla, Ariel Gianecini, Mónica Prieto, Marcelo Gottschalk, Nahuel Fittipaldi

**Affiliations:** Author affiliations: Université de Montréal Faculté de médecine vétérinaire, St-Hyacinthe, Quebec, Canada (M.V.D.P. Bakpatina-Batako, K. Li, S. Lacouture, M. Gottschalk, N. Fittipaldi); Instituto Nacional de Enfermedades Infecciosas, ANLIS Dr. Carlos G. Malbrán, Buenos Aires, Argentina (L. Cipolla, A. Gianecini, M. Prieto)

**Keywords:** Streptococcus suis, Streptococcus parasuis, zoonoses, bacteria, antimicrobial resistance, South America, Argentina, Brazil, Chile, French Guiana, Uruguay, whole-genome sequencing

## Abstract

*Streptococcus suis*, a swine pathogen that causes zoonotic infections in Europe and Asia, has increasingly been observed in South America. We reviewed all available reports from the continent and identified *S. suis* cases in Argentina, Brazil, Chile, French Guiana, and Uruguay. We also identified 8 novel infections from Argentina, bringing the total documented human cases in South America to 47. We reclassified 1 previously reported infection as *S. parasuis*. Among the 47 *S. suis* cases, 40 (85%) patients had meningitis, 2 (4%) had toxic shock–like illness, 2 (4%) had nonshock sepsis, 1 (2%) had arthritis, and 1 (2%) had endocarditis. The case-fatality rate was 4% (2/47). Infections were primarily linked to pig or pork exposure, although some occurred after consuming undercooked meat. Case distribution varied by country, and Argentina reported a disproportionately high number of cases despite a smaller swine industry. Our findings highlight the need for more consistent regional *S. suis* surveillance.

*Streptococcus suis* is a swine pathogen and a zoonotic agent responsible for meningitis, septicemia, streptococcal toxic shock–like syndrome (STSLS), and other diseases in humans, particularly among persons who have close contact with pigs or pork by-products or who consume dishes made with raw pork or pig blood (*1*,*2*). *S. suis* is phenotypically and genetically highly diverse, has 29 recognized serotypes on the basis of serologic reactions against its polysaccharide capsule, and has >2,900 sequence types (STs) defined by multilocus sequence typing (MLST) (*3*,*4*). Resistance to macrolide, lincosamide, and tetracycline antibiotics is common, and recent reports indicate emerging β-lactam resistance (*5*).

Most human infections are caused by *S. suis* serotype 2, although cases caused by serotypes 1, 4, 5, 7, 9, 14, 16, 21, 24, and 31 have been reported (*6*,*7*). Most human infections have been reported in East and Southeast Asia (*2*). In Europe, *S. suis* is considered an occupational disease, and human infections are less frequently reported than in Asia but still relatively common (*8*). Sporadic *S. suis* cases have also been documented in Hawaii (USA), Australia, Togo, and Madagascar (*9*–*12*), but <10 cases have been described from the continental United States and Canada (*13*), despite the large swine farming industries in those regions. Reports from South America have been less integrated into the global understanding of *S. suis* zoonotic disease, partly because many were not published in English. To address that gap, we reviewed published cases from South America, identified and report on novel human infections from Argentina, and assessed epidemiologic and clinical characteristics of *S. suis* infections on the continent.

## Materials and Methods

### Literature Review

To identify reported cases of human *S. suis* infections in South America, we conducted a comprehensive literature review as a critical narrative synthesis rather than a systematic review or meta-analysis. We included PubMed, ScienceDirect, SciELO (Scientific Electronic Library Online), and Google Scholar databases, as well as gray literature, by using the following key terms: *S. suis*, South America, human infection, meningitis, sepsis, zoonosis, pig, pork, and the names of all countries in South America. We conducted searches in English, Dutch, French, Portuguese, and Spanish. We also reviewed PubMLST (https://pubmlst.org) and GenBank to identify potentially unreported human cases. We selected studies and case reports on human *S. suis* from South America, excluded duplicates or reports involving isolates from outside the continent, and extracted relevant patient and causative-isolate metadata. Through the literature review, we identified 39 confirmed *S. suis* human cases in South America during 1995–2024 (*14*–*28*). We also noted a potential additional case on the basis of a human isolate submitted to PubMLST.

### Data Collection and Microbiology Methods for Previously Undescribed Cases from Argentina

In addition to cases identified by reviewing the literature, we report 8 previously undescribed *S. suis* human infections diagnosed and microbiologically confirmed during 2017–2024 through national laboratory surveillance in Argentina, bringing the total *S. suis* cases in the continent to 47. For the 8 new cases, we collected clinical data including demographic details, risk factors, diagnostic methods, treatment, and outcomes from patient medical records at hospitals participating in the Argentine National Laboratory Network for Meningitis and Invasive Bacterial Diseases, which does not require ethics approvals to share anonymized patient data and isolates with the Instituto Nacional de Enfermedades Infecciosas, ANLIS Dr. Carlos G. Malbrán, where the data were centralized. The same hospitals submitted the causative agent associated with those cases (i.e., the α-hemolytic gram-positive isolates identified as *S. suis* through biochemical testing) to the reference laboratory. Species confirmation was carried out via matrix-assisted laser desorption/ionization time-of-flight mass spectrometry and by PCR targeting the species-specific *recN* gene (*29*). Serotyping was performed using the co-agglutination test (*30*), a multiplex PCR targeting genes in the *S. suis* capsular polysaccharide (*cps*) locus (*31*), or both. To differentiate between *S. suis* and *S. parasuis*, we performed an in silico PCR targeting the *recN* gene using primers from an established identification scheme (*32*).

### Whole-Genome Sequencing and Analysis

We prepared genomic libraries for the 8 new and 1 previously reported *S. suis* isolates from Argentina by using Nextera XT kits (Illumina, https://www.illumina.com) and sequenced them on an Illumina MiSeq as 150-bp paired end reads. We deposited data into the National Center for Biotechnology Information Sequence Read Archive (https://www.ncbi.nlm.nih.gov/sra) (Appendix Table 1). We determined or confirmed speciation, serotyping, MLST profiles, and antimicrobial resistance (AMR) gene content by using the short-read genomic data and an *S. suis* typing pipeline (*33*), SRST2 software (*34*), and the Comprehensive Antibiotic Resistance Database version 3.0.8 (*35*).

### Phylogenetic Analysis

We used Snippy (https://github.com/tseemann/snippy) to identify single-nucleotide polymorphisms in the genome of the isolates (Table 1) relative to the genome of the ST1 serotype 2 *S. suis* reference strain P1/7 (GenBank accession no. NC_012925.1). We used the Snippy-core function to define core-genome single-nucleotide polymorphisms, which we used to build a maximum-likelihood phylogenetic tree in FastTree 2.1.10 (*37*) with Shimodaira–Hasegawa–like local support values based on 1,000 resamples. We visualized and annotated trees in R (The R Project for Statistical Computing, https://www.r-project.org) by using the ggtree library (*38*).

**Table 1:** Summary of human *Streptococcus suis* infections, South America, 1995–2024*

Patient characteristics	Argentina	Brazil	Chile	French Guiana	Uruguay	South America
Total no. cases	29	5	7†	1	5	47
Sex						
M	25	5	3	1	4	38
F	4	0	2	0	0	6
Not reported	0	0	2	0	1	3
Age, y						
Mean age, y (range)‡	46.6 (38–55)	65.6 (50–82)	49.6 (44–55)	42 (NA)	55 (50–66)	48.5 (38–82)
Median age, y‡	47	68	48	NA	52	50
Not reported	20	0	2	0	1	23
Clinical manifestation						
Meningitis	26	4	5	1	4	40
STSLS	1	1	0	0	0	2
Other§	2	0	2	0	0	4
Not known	0	0	0	0	1	1
Outcomes						
Recovered	27	5	5	1	5	43
Died	2	0	0	0	0	2
Hearing loss	1	3	1	1	1	7
Not known	0	0	0	0	1	1
Contact with pigs						
Worker or farmer	18	3	2	1	4	28
Other contact¶	4	2	1	0	0	7
No contact	7	0	0	0	0	7
Not known	0	0	4	0	1	5
Isolates						
Serotyping						
Reported#	26	1	7	1	0	35
Not reported	3	4	0	0	5	12
MLST						
Reported**	25	0	0	1	0	26
Not reported	4	5	7	0	5	21

*MLST, multilocus sequence type; ST, sequence typing; STSLS, streptococcal toxic shock-like syndrome. †Because 1 human isolate from Chile was available in PubMLST, an additional human case is possible. That isolate belonged to MLST ST1172, but it was not serotyped. Thus, whether that isolate represents an eighth human infection in Chile or if it corresponds to one of the 7 previously reported infections is not known. ‡One case occurring in a 1-y-old infant from Argentina was excluded when calculating mean, range, and median patient age. §Includes 1 case of septic arthritis and 1 case of endocarditis in Argentina and 2 cases of sepsis in Chile. ¶Includes contact while handling pigs for consumption, consuming undercooked pork, hunting wild boars, or a combination of those factors. #All cases where serotyping was performed revealed serotype 2 isolates except for 1 serotype 5 and 1 untypable isolate in Argentina. **All cases where MLST was performed revealed ST1 isolates, except for an ST5 strain that was retrospectively ascribed to ST483 in a different investigation (*36*).

## Results

### Epidemiology of Human *S. suis* Infections in South America

Including the 8 novel *S. suis* infection we report here, we found a total of 47 confirmed *S. suis* human cases in South America during 1995–2024 (*14*–*28*). We also noted a potential additional case from a human isolate submitted to PubMLST. Most (n = 38) patients were adult or older adult men; 6 cases were among female patients, including 1 infant, and sex was unreported for 3 cases (Table 1). Forty (85%) patients had meningitis, and 7 of those patients experienced permanent or temporary hearing loss. The other 7 cases involved severe sepsis and STSLS (2 each) and septic arthritis and endocarditis (1 each); 1 case had no available diagnosis (Table 1). Only 2 deaths were reported, a fatality rate of 4%. Contact with pigs or pig products was a common risk factor: 28 patients were workers or farmers with direct exposure, and 7 patients reported contact through handling, exposure to, or consuming swine or wild boar. Exposure information was unavailable for 5 cases, and 7 persons reported no known exposure (Table 1).

### Previously Reported *S. suis* Human Infections in Argentina

Before our analysis, Argentina had documented a total of 21 *S. suis* infections (*14*–*18*) (Appendix Table 2). The first reported case, in 2005, involved a female patient in whom bacterial meningitis developed after occupational pig exposure (*18*). The isolate, identified as *S. suis* serotype 2 with the muramidase released protein (*mrp*), extracellular protein factor (*epf*), and suilysin (*sly*) (*mrp/epf/sly*) virulence gene profile typical of strains from Eurasia, was later typed by MLST as ST1 (*14*,*18*). A second report, from 2006, described a 49-year-old man from Santa Fe Province with meningitis who had occupational exposure at a slaughterhouse (*15*). That patient was treated with ampicillin and ceftriaxone, initially improved, but later had complete hearing loss in the left ear and partial loss in the right develop. Cerebrospinal fluid (CSF) cultures grew *S. suis*, but the isolate was not typed. Another report described a 54-year-old man from a rural area of Tucumán Province with meningitis who had occupational exposure to pigs (*16*). *S. suis* was isolated from CSF, but the strain was not typed. He was treated with ceftriaxone and dexamethasone and recovered without long-term neurologic sequelae.

Those 3 cases, along with a fourth potential human infection with *S. suis* serotype 21 (*39*), reclassified in this investigation as *S. parasuis*, prompted the retrospective investigation of 17 additional infections, including 5 involving male patients with meningitis that predated previous reports, 2 from 1995 and 3 from 2003. The other cases spanned 2009–2016 and involved 10 male and 2 female patients, mostly from rural areas (*14*). Of those 17 cases, 12 involved direct contact with pigs or pork products, typically through farming or slaughterhouse work; 1 had no reported contact, and 4 lacked exposure data. Sixteen cases had meningitis as the clinical manifestation, and 1 involved septic arthritis (*14*). The infections were all caused by *S. suis* ST1 serotype 2, except for a 2014 case involving serotype 5, which was the only fatality in this series (*14*).

In 2024, a 42-year-old man working at a pig processing plant in Argentina was hospitalized with meningitis after 10 days of weakness, headache, progressive hearing loss, and ataxia. CSF cultures grew *S. suis* serotype 2. He was treated with ceftriaxone and dexamethasone and improved clinically, but mild bilateral hearing loss persisted (*17*).

### New *S. suis* Human Infections in Argentina, 2017–2024

We identified 8 additional, previously unreported *S. suis* infections from Argentina that occurred during 2017–2024 (Table 2). Case 1, from 2017, involved a 42-year-old male pig farmer who had fever, fatigue, and shortness of breath. Blood cultures grew *S. suis*, but the isolate was untypable. Further investigation revealed native valve endocarditis. The patient recovered without complications.

**Table 2:** Characteristics of patients and isolates for the 8 new cases of human *Streptococcus suis* infection from Argentina, South America, 2017–2024*

Year	Patient						Isolate				
	Age, y/sex	Disease	Contact with swine or pork	Outcome	Notes		Isolate ID	Source	Serotype	ST	Virulence markers
2017	42/M	NVE	Yes, pig farmer	Survived	None		26-17	Blood	Untypable†	1	*mrp*,* epf*,* sly*	
2018	1/F	Meningitis	Unknown, lived in rural area	Survived	None		868-18	Blood	2	1	*mrp*,* epf*,* sly*	
2019	Adult/M‡	Meningitis	Unknown	Survived	None		724-19	CSF	2	1	*mrp*,* epf*,* sly*	
2020	38/M	Meningitis	No, lived in urban area	Survived	Inhalation drug user		247-20	Blood, CSF§	2	1	*mrp*,* epf*,* sly*	
2021	47/M	Meningitis	Yes, consumed pork	Survived	Wild boar hunter		521-21	Blood, CSF§	2	1	*mrp*,* epf*,* sly*	
2022	48/M	Meningitis	Yes, consumed pork	Survived	Wild boar hunter		395-22	CSF	2	1	*mrp*,* epf*,* sly*	
2022	45/M	Meningitis	Yes, handled not commercial pig	Survived	None		931-22	CSF	2	1	*mrp*,* epf*,* sly*	
2024	55/M	STSLS	Yes	Died	None		649-24	Blood	2	1	*mrp*,* epf*,* sly*	

*CSF, cerebrospinal fluid; *epf *extracellular protein factor; ID, identification; *mrp*, muramidase released protein; NVE, native valve endocarditis;* sly*, suilysin; ST, sequence type as defined by multilocus sequence typing; STSLS, streptococcal toxic shock–like syndrome. †Untypable by both serologic and molecular methods. ‡Precise age not available. §Only the isolate recovered from CSF was included in this investigation, although both were typed.

In Case 2, from 2018, a 1-year-old girl from a rural area was admitted to the hospital with signs of meningitis. Blood cultures grew *S. suis* serotype 2. The patient survived the infection. Exposure to pigs or pork could not be determined, and no household pig-keeping or sick contacts were reported.

Case 3, from 2019, involved an adult man with meningitis, but the source of exposure could not be determined. CSF cultures grew *S. suis* serotype 2. The patient survived and did not have residual sequelae.

Case 4 occurred in 2020 and involved a 38-year-old man with meningitis who was from an urban area and used drugs but had no reported contact with pigs or pork products. Blood and CSF cultures grew *S. suis* serotype 2. The patient survived without long-term complications.

Case 5 was identified in 2021 in a 47-year-old male hunter in whom meningitis developed after contact with wild boars and consuming a suckling pig. Blood and CSF cultures grew *S. suis* serotype 2. The patient survived without notable sequelae.

Case 6, from 2022, was in a 48-year-old man who regularly hunted wild boars and had meningitis develop after consuming pork. CSF cultures grew *S. suis* serotype 2. The patient survived without notable sequelae.

Case 7 was reported in 2022 in a 45-year-old man who had meningitis after purchasing and preparing a pig from a backyard farm. CSF cultures grew *S. suis* serotype 2. The patient survived without notable sequelae.

Finally, case 8 occurred in 2024 in a 55-year-old man in whom STSLS developed after confirmed contact with pigs. Blood cultures grew *S. suis* serotype 2. Despite aggressive treatment, the patient died, the only fatality in this 8-case series.

### *S. suis* Human Infections in Argentina and Reclassification of 1 Isolate as *S. parasuis*

An unusual infection attributed to the rare *S. suis* serotype 21 was described in 2014 in Argentina (*39*). The patient had spontaneous bacterial peritonitis (*39*), raising questions about the initial classification, especially because co-agglutination, the serologic typing method initially used, can sometimes produce false cross-reactive results (*30*). To resolve those inconsistencies, we re-examined the isolate using genomic data. An ad hoc in silico PCR with specific primers targeting the *recN* gene of various streptococcal species reclassified the isolate as *S. parasuis*, a species closely related to *S. suis* that was not formally recognized until 2015 (*29*), after the initial erroneous identification in 2014. Further analysis corroborated that reclassification (Appendix Figure). That case predates similar cases of human *S. parasuis* infection reported in China (*40*).

### *S. suis* Human Infections in Brazil

In Brazil, 5 human *S. suis* infections have been documented, involving both occupational exposure and ingesting undercooked pork (*19*–*22*) (Appendix Table 2). The first, which occurred in 2019 but was reported in 2024, involved a 68-year-old swine farmer from Bahia who had bacterial meningitis. CSF cultures grew *S. suis*, but serotyping was not performed. The patient experienced permanent hearing loss (*21*). In 2020, two additional cases were reported in Ceará, both involving men with occupational exposure to pigs who had meningitis. Serotyping of the isolates was not performed in either of those cases (*20*).

The other 2 cases from Brazil involved patients who reported consuming pork. The first, in 2020, involved an 82-year-old man from Rio de Janeiro who contracted *S. suis* meningitis after consuming pork that probably was undercooked; the specific serotype was not reported (*19*). The second, in 2024, involved a 50-year-old man from São Paulo who had STSLS and probably meningitis after consuming raw pork. Blood cultures confirmed *S. suis* serotype 2. Although that patient recovered, he had hearing loss develop (*22*).

### *S. suis* Human Infections in Chile

In Chile, 7 human *S. suis* infections have been reported, all linked to occupational or incidental exposure to pigs (*23*–*25*) (Appendix Table 2). The first 2 cases, reported in 2012, involved a 54-year-old woman and a 48-year-old man, both pig farmers who had bacterial meningitis. CSF cultures grew *S. suis* serotype 2. Both patients recovered without neurologic sequelae (*23*,*24*). In 2013, two more cases were reported, both involving men who had meningitis or sepsis caused by *S. suis* serotype 2 (*23*). In 2014 and 2015, two additional cases were documented in Región de los Ríos: 1 patient had severe sepsis, the other meningitis, and serotypes were not reported (*25*). A seventh case occurred in 2018, involving a 44-year-old woman in whom meningitis developed 2 days after handling raw pork and who experienced permanent bilateral hearing loss postinfection. *S. suis* serotype 2 was confirmed through CSF cultures (*25*). We suspect a potential eighth human case in Chile on the basis of a 2019 submission of a human ST1172 *S. suis* isolate to PubMLST; whether that isolate represents a new infection or a previously documented case is unclear.

### *S. suis* Human Infections in French Guiana.

In French Guiana, a single *S. suis* human infection was reported in 2011 (*26*) (Appendix Table 2). In that case, meningitis developed in a 42-year-old man originally from Haiti who injured his thumb while slaughtering pigs. CSF cultures grew *S. suis* ST1 serotype 2. He experienced moderate bilateral hearing loss, which later progressed to severe hearing loss in the left ear (*26*).

### *S. suis* Human Infections in Uruguay

Five human *S. suis* infections have been reported in Uruguay (*27*,*28*) (Appendix Table 2). The first 2 cases were identified in 2008 and 2009 in men who had occupational exposure to pigs and had acute meningitis (*28*), 1 of whom experienced bilateral hearing loss. A third case was reported in Paysandú in 2009; the patient had similar occupational exposure and clinical features (*28*). Fifteen years later, 2 additional cases were reported in men who worked in rural areas (*27*). Both patients had meningitis develop after handling pigs and were treated successfully, but 1 patient had mild neurologic symptoms that persisted. None of the *S. suis* isolates responsible for human infections in Uruguay were serotyped.

### Characteristics of *S. suis* Isolates from Human Infections in South America

Of the 47 confirmed and 1 potential human *S. suis* infections in South America, only 35 isolates were serotyped. Serotype 2 was identified in all but 2 isolates, 1 of which was serotype 5 and the other was untypable (Table 1). The serotype 5 isolate was retrospectively assigned to ST486 (*36*). The other isolates with available serotype and MLST data were either ST1 serotype 2 (n = 25) or untypable (n = 1). 

We saw notable differences in isolate investigation among countries. The French Guiana isolate was typed as ST1 serotype 2. In Argentina, serotype was available for 26 isolates, 25 of which also had MLST data. Most isolates from Chile were serotyped but not MLST typed, except for 1 that was typed as ST1172, a close derivative of ST1, but was not serotyped. None of the 10 isolates from Uruguay and Brazil were MLST typed, and only 1 isolate from Brazil was serotyped (Table 1).

Genome data for *S. suis* isolates from South America were limited to 9 previously reported isolates from human infections in Argentina (*14*) and 8 newly sequenced genomes from our study. Seeking broader representation, we contacted authors of previous reports from other countries in South America. However, we could not obtain additional isolates because of unavailability of isolates or lack of timely responses from authors. 

**Figure 1:**
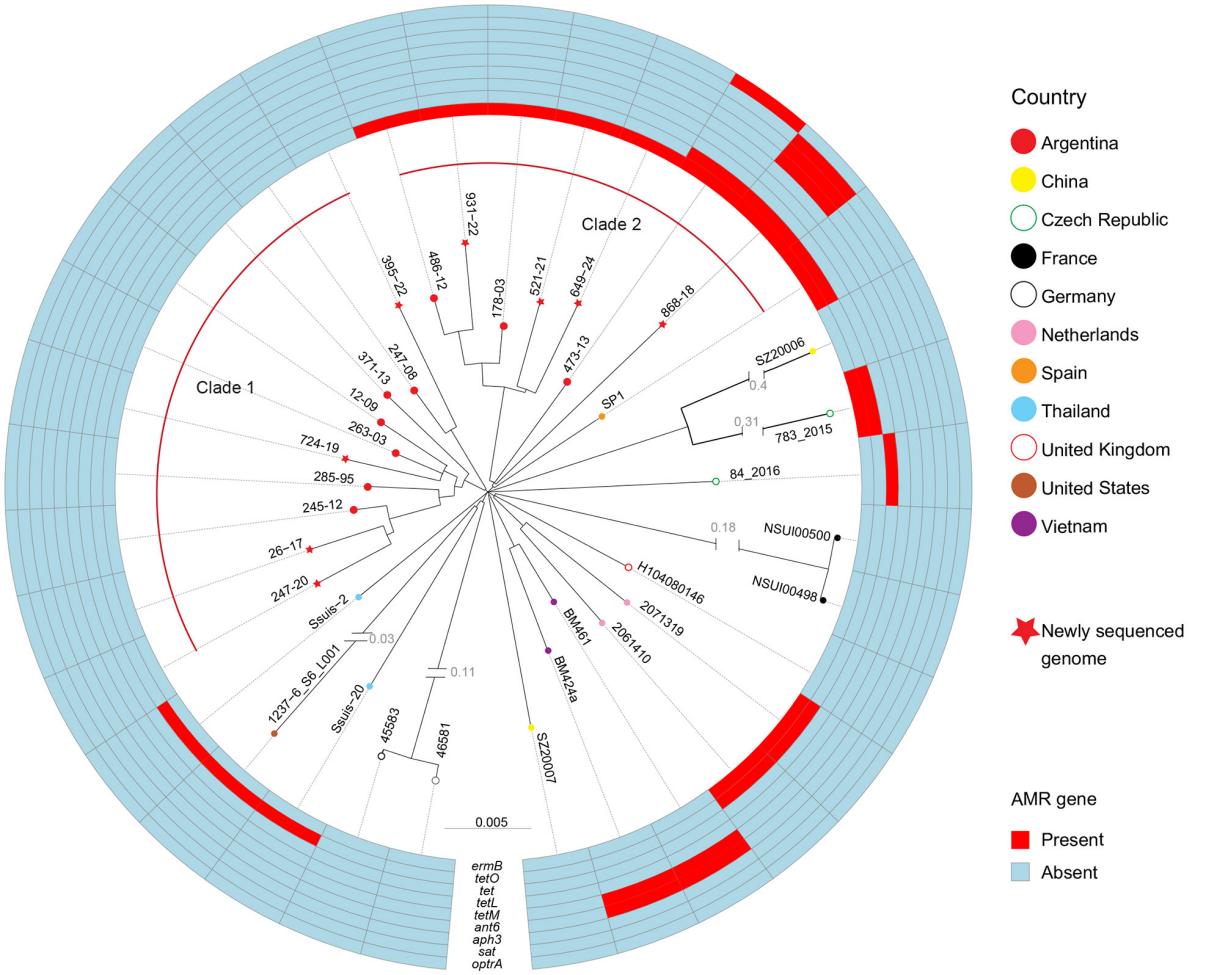
Phylogenetic relationships and AMR gene profiles of isolates in a study of human *Streptococcus suis* infections, South America, 1995–2024. Maximum-likelihood phylogenetic tree shows relationships among sequence type (ST) 1 serotype 2 *S. suis* isolates from human disease in Argentina and selected ST1 serotype 2 isolates from other countries. Tree was constructed by using 5,504 bp nonredundant core-genome single-nucleotide polymorphism loci identified relative to the genome sequence of ST1 serotype 2 reference strain P1/7 (not included in the depiction). The tree delineates 2 distinct clades from Argentina that differ on AMR gene content. One isolate from a human infection in Spain clusters tightly within clade 2 from Argentina, but the rest of the isolates, all from outside South America, are more distantly related genetically to either clade from Argentina. AMR genes identified in the genomes of the isolates are represented in the outer rings. AMR, antimicrobial resistance.

Because all available genomes originated from Argentina, we expanded the phylogenetic analysis by incorporating ST1 isolates from other geographic regions (Appendix Table 1). Phylogenetic analysis revealed that the isolates from Argentina clustered into 2 distinct clades (Figure 1), both relatively distantly related to ST1 isolates from other regions, except for 1 isolate from Spain that clustered tightly with isolates of 1 of the Argentina subclades. All isolates from Argentina had an *mrp*/*epf*/*sly* virulence marker profile and multiple AMR genes identified, but the AMR genes were restricted to a single clade (Figure 1).

## Discussion

Because of the small sample size, uneven subgroup distribution, and missing data for key variables, our dataset did not support formal statistical analysis of associations between demographic or exposure-related factors and outcomes; therefore, we focused on descriptive patterns. As expected, based on current *S. suis* knowledge (*2*,*4*,*8*), adult men whose occupations involved direct contact with pigs or pork products were the primary at-risk population in South America. Raw pork consumption, an established risk factor for *S. suis* transmission in parts of Southeast Asia, is uncommon in South America, where pork is typically roasted or stewed (*2*,*14*). Nonetheless, at least 3 cases in South America occurred in persons who had consumed home-prepared suckling pig or wild boar meat that might have been undercooked, highlighting foodborne transmission as the plausible route of infection.

Among the entire cohort, meningitis was the most common manifestation, and numerous cases of subsequent hearing loss were reported, likely resulting from cochlear and labyrinth damage (*2*,*41*–*44*). Early audiometric evaluation is recommended to promptly manage hearing impairment in such cases (*43*). Predominance of meningitis likely reflects its more rapid recognition in hospital settings (*43*). Similarly, severe symptoms associated with STSLS demand immediate medical attention and follow up (*45*). Conditions like arthritis and endocarditis, though, may be underdiagnosed or misattributed to other pathogens because of low awareness and outdated biochemical methods. Those factors often lead to misidentifying *S. suis* as other streptococci, such as viridans group *Streptococcus*, even in high-income countries (*2*,*13*,*46*). Training healthcare professionals to consider *S. suis* when α-hemolytic streptococci are isolated and to take detailed histories of pig or pork contact is essential. In addition, implementing referral protocols and using advanced matrix-assisted laser desorption/ionization time-of-flight mass spectrometry and molecular diagnostic testing can improve the detection and diagnosis of *S. suis* infections.

**Figure 2:**
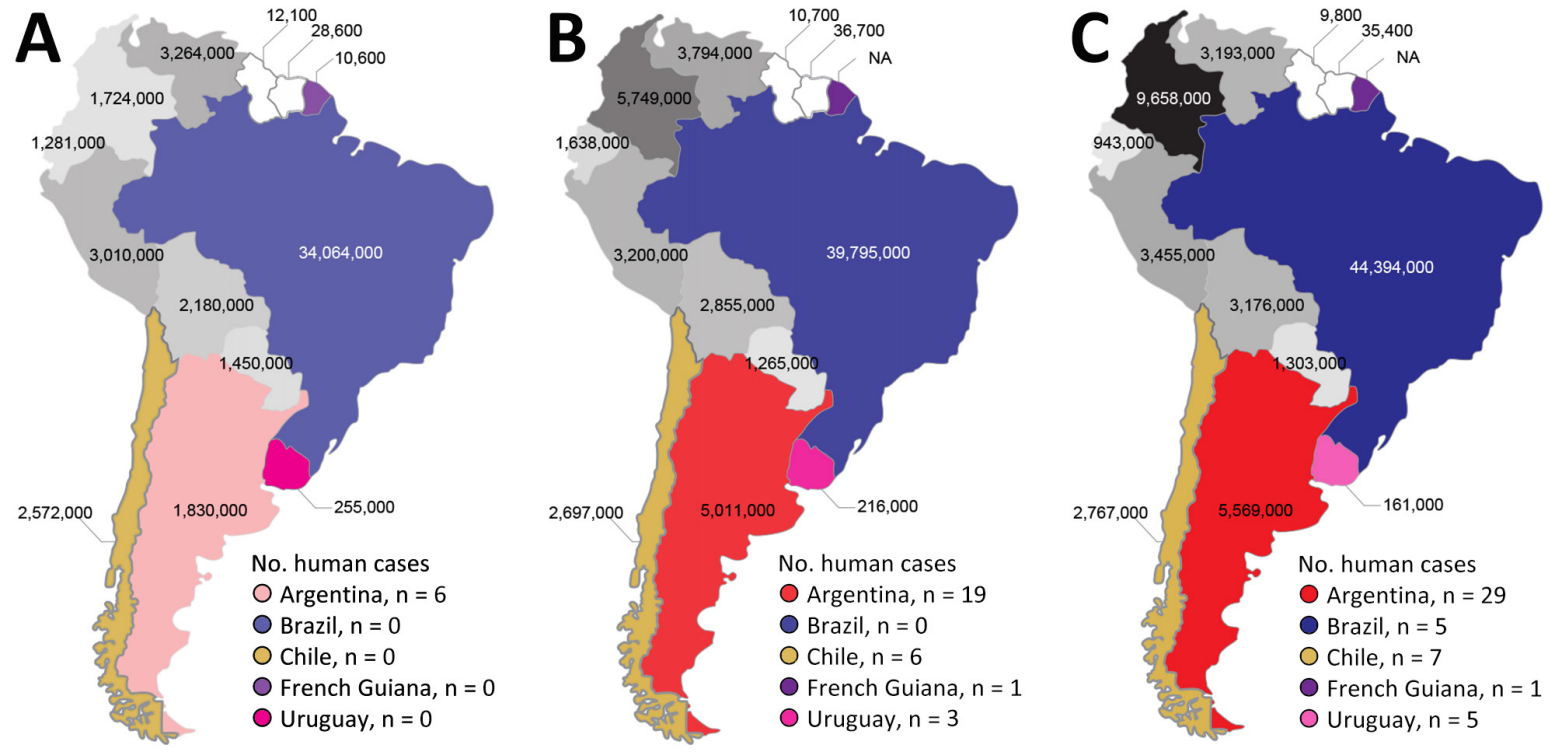
Estimated number of swine and cumulative reported human cases per country in a study of human *Streptococcus suis* infections, South America, 1995–2024. Shown are countries with human *S. suis* infections reported during 1995–2005 (A), 1995–2015 (B), and 1995–2024 (C). Estimated number of heads of swine is shown for each country from 2005 (A), 2015 (B), and 2022 (C). Countries with reported human *S. suis* infections are depicted in specific colors, and cumulative number of human cases during each timeframe are provided in each key. The color intensity increases proportionally to reflect changes in swine population size over time in each country. In contrast, countries without reported human *S. suis* infections are represented in grayscale, and the grayscale intensity indicates variations in swine population size, applied on a unified scale across those countries. Swine production data for French Guiana was only available until 2006. NA, not available.

The number of reported human *S. suis* infections in South America does not correlate with the size of national swine industries (Figure 2). Brazil, a global leader in pork production with >40 million pigs, has reported only 5 human cases. In contrast, Argentina, with a much smaller swine population of 5 million, has documented 29 infections. Biosecurity, as traditionally defined in swine production, has not been shown to reduce *S. suis* disease in pigs or the associated zoonotic risk in humans; nonetheless, the structured occupational health protocols in Brazil’s large-scale farms might play a role in limiting human exposure (*47*). In Argentina, the swine industry has expanded rapidly in the past 2 decades, and the number of pigs more than doubled from 2005 to 2015 (Figure 2). However, small-scale farms with limited implementation of biosafety practices remain common and may be contributing to the higher number of reported infection rates (*14*,*47*). Similarly, Uruguay has a small swine industry dominated by extensive farming (*48*) and reported 5 human cases during the study period. In Chile, most reported human cases are linked to traditional small-scale farming practices, even though the cases are geographically concentrated in the south of the country, where export-driven large-scale swine operations are located (*47*,*49*).

No human *S. suis* infections have been reported from other South America countries, including Colombia, which has a swine industry larger in scale and comparable in growth to Argentina’s (*47*) (Figure 2). Thus, factors beyond the scale and type of pig farming likely influence the incidence of this zoonotic disease. We speculate that differences in the virulence of circulating *S. suis* strains likely play a role. For example, less virulent ST25 and ST28 serotype 2 strains are predominant in North America, which reports very few human infections, but highly virulent ST1 serotype 2 strains are common in Eurasia (*4*,*13*). In Argentina, highly virulent ST1 serotype 2 strains are prevalent, and ST1 or closely related ST1172 isolates have been identified in French Guiana and Chile. It may be possible that strains of lower virulence circulate among swine in Brazil, Colombia, and other countries that have few or no reported human cases. However, the paucity of molecular data on *S. suis* isolates from most countries in South America, even from pigs, hinders the ability to confirm that hypothesis. Furthermore, our retrospective reclassification of 1 misidentified isolate as *S. parasuis* highlights the value of genomic methods for accurate species identification and for advancing our understanding of *S. suis* disease epidemiology.

Another, perhaps more plausible, explanation for the higher number of reported cases in Argentina compared with other countries in South America is the well-established surveillance system for α-hemolytic bacteria associated with severe infections, particularly in rural hospitals. Suspicious isolates are systematically referred to the National Institute of Infectious Diseases for confirmation and detailed identification, enabling more accurate detection and reporting of *S. suis* infections (*14*). That systematic approach likely accounts for the relatively higher detection rates in Argentina. Enhancing surveillance systems and laboratory capacities in other countries in South America could similarly improve case identification, support more consistent and standardized reporting of demographic and clinical data, and ultimately enable more robust epidemiologic analyses across the continent.

Our findings highlight a complex interplay of factors influencing the incidence and reporting of human *S. suis* infections in South America. Reported cases tend to be more frequent in countries where small-scale or mixed farming persists, reflecting the increased risk for zoonotic transmission under those conditions. Close human–pig interactions and limited on-farm biosafety measures likely contribute to that risk. The confirmed presence of highly virulent ST1 serotype 2 strains in Argentina and the robust surveillance system in that country likely contribute to its higher detection rates and detailed epidemiologic data.

In summary, addressing *S. suis* human infections requires a coordinated approach, including improved on-farm biosafety practices, particularly among small-scale farmers, and strengthened surveillance systems. Argentina’s proactive efforts could serve as a model for enhancing diagnostic capabilities across the region. International collaboration, data sharing, and partnerships with the swine industry are essential to better characterize *S. suis* strains and reduce the burden of this zoonotic disease in South America.

Appendix Additional information on human *Streptococcus suis* infections, South America, 1995–2024.
