# Supplementary material for: Human Streptococcus suis Infections, South America, 1995–2024
Source: Emerg Infect Dis. 2025 Jul;31(7):1277–86. doi: 10.3201/eid3107.241835 (PMC12205450; doi:10.3201/eid3107.241835)
Supplement: Appendix — Additional information on human Streptococcus suis infections, South America, 1995–2024. [file 24-1835-Techapp-s1.pdf]

Article DOI: https://doi.org/10.3201/eid3107.241835

EID cannot ensure accessibility for supplementary materials supplied by authors. Readers who have difficulty accessing supplementary content should contact the authors for assistance.

Human *Streptococcus suis* Infections, South America, 1995–2024

Appendix

Appendix Table 1. *Streptococcus suis* isolates included in phylogenetic analysis*

Isolate ID	Country	Year	Host	Serotype	Sequence type†	SRA accession no.‡
649-24§	Argentina	2024	Human	2	1	SRR31573597
395-22§	Argentina	2022	Human	2	1	SRR31573596
931-22§	Argentina	2022	Human	2	1	SRR31573595
521-21§	Argentina	2021	Human	2	1	SRR31573594
247-20§	Argentina	2020	Human	2	1	SRR31573593
724-19§	Argentina	2019	Human	2	1	SRR31573592
868-18§	Argentina	2018	Human	2	1	SRR31573591
26-17§	Argentina	2017	Human	Untypable	1	SRR31573590
473-13	Argentina	2013	Human	2	1	SRR3954843
371-13	Argentina	2013	Human	2	1	SRR3954842
486-12	Argentina	2012	Human	2	1	SRR3954846
245-12	Argentina	2012	Human	2	1	SRR3954845
12-09	Argentina	2009	Human	2	1	SRR3954844
178-03	Argentina	2003	Human	2	1	SRR3954841
247-08	Argentina	2003	Human	2	1	SRR3954839
263-03	Argentina	2003	Human	2	1	SRR3954837
285-95	Argentina	1995	Human	2	1	SRR3954838
SZ20006	China	2000	Human	2	1	SRR22062755
SZ20007	China	2000	Human	2	1	SRR22062754
783_2015	Czech Republic	2015	Human	2	1	SRR26047317
84_2016	Czech Republic	2016	Human	2	1	SRR26047316
NSUI00498	France	NA	Pig	2	1	SRA40946613
NSUI00500	France	NA	Pig	2	1	SRA40946615
45583	Germany	2010	Human	2	1	SRR26047304
46581	Germany	2010	Human	2	1	SRR26047303
2061410	Netherland	2006	Human	2	1	ERR1055576
2071319	Netherland	2007	Human	2	1	ERR1055577
SP1	Spain	2014	Human	2	1	SRR26047283
Ssuis-2	Thailand	2007	Human	2	1	SRR10387933
Ssuis-20	Thailand	2007	Pig	2	1	SRR10387932
H104080146	United Kingdom	2010	Human	2	1	SRR26047285
1237–6_S6_L001	United States	2019	Pig	2	1	SRR24624552
BM424a	Vietnam	2004	Human	2	1	ERR193254
BM461	Vietnam	2014	Human	2	1	ERR193260

*ID, identification; SRA, NCBI's Sequence Read Archive.
†Sequence type as determined by multilocus sequence typing.
‡Note that in this study we also sequenced the genome of a *Streptococcus parasuis* human isolate (1368610), whose SRA accession number is SRR31573589.
§Indicates isolates whose genomes were sequenced in this study.

Appendix Table 2. *Streptococcus suis* human infections previously reported in South America*

		Patient characteristics					Isolate			
Year	Country	Age	Sex	Contact with swine	Disease manifestation	Outcome	Isolate ID	Serotype	ST	Reference
1995	Argentina	NR	M	Yes	Meningitis	Recovered	285	2	1	(*1*)
1995	Argentina	NR	M	Yes	Meningitis	Recovered	284	2	1	(*1*)
2003	Argentina	NR	M	Yes	Meningitis	Recovered	263	2	1	(*1*)
2003	Argentina	NR	M	Yes	Meningitis	Recovered	178	2	1	(*1*)
2003	Argentina	NR	M	Yes	Meningitis	Recovered	247	2	1	(*1*)
2004	Argentina	NR	F	Yes	Meningitis	Recovered	2376	2	1	(*2*)
2006	Argentina	49	M	Yes	Meningitis	Hearing loss	NR	NR	NR	(*3*)
2009	Argentina	NR	M	Yes	Meningitis	Recovered	12	2	1	(*1*)
2009	Argentina	NR	M	NR	Meningitis	Recovered	83	2	1	(*1*)
2009	Argentina	NR	M	Yes	Meningitis	Recovered	88	2	1	(*1*)
2012	Argentina	NR	M	Yes	Meningitis	Recovered	245	2	1	(*1*)
2012	Argentina	NR	M	NR	Meningitis	Recovered	486	2	1	(*1*)
2013	Argentina	NR	M	Yes	Meningitis	Recovered	371	2	1	(*1*)
2013	Argentina	NR	F	NR	Meningitis	Recovered	473	2	1	(*1*)
2013	Argentina	54	M	Yes	Meningitis	Recovered	NR	NR	NR	(*4*)
2013†	Argentina	62	M	No	Peritonitis	Recovered	1368610	21†	NR	(*5*)
2014	Argentina	NR	F	NR	Meningitis	Recovered	42	2	1	(*1*)
2014	Argentina	NR	M	Yes	Meningitis	Died	15	5	486	(*1*,*6*)
2015	Argentina	NR	M	Yes	Meningitis	Recovered	130/15	2	1	(*1*)
2015	Argentina	NR	M	Yes	Meningitis	Recovered	695-15	2	1	(*1*)
2016	Argentina	NR	M	Yes	Arthritis	Recovered	136-16	2	1	(*1*)
NR‡	Argentina	42	M	Yes	Meningitis	Recovered	NR	NR	NR	(*7*)
2019	Brazil	68	M	Yes: Farmer, worker	Meningitis	Hearing Loss	NR	NR	NR	(*8*)
2020	Brazil	60	M	Yes: Farmer, worker	Meningitis	Hearing Loss	NR	NR	NR	(*9*)
NR	Brazil	82	M	Yes: Consumption of undercooked pork	Meningitis	Recovered	NR	NR	NR	(*10*)
2020	Brazil	68	M	Yes: Farmer Worker	Meningitis	Recovered	NR	NR	NR	(*9*)
2024	Brazil	50	M	Yes: Consumption of undercooked pork	STSLS; probable meningitis§	Hearing Loss	NR	2	NR	(*11*)
2012	Chile	54	F	Yes: Farmer worker	Meningitis	Recovered	CL-APA-SSU-001	2	NR	(*11*)
2012	Chile	48	M	Yes: Farmer worker	Meningitis	Hearing Loss	CL-APA-SSU-002	2	NR	(*11*)
2012	Chile	55	M	NR	Meningitis	Recovered	CL-APA-SSU-003	2	NR	(*12*)
2013	Chile	47	M	NR	Septicemia	Recovered	CL-APA-SSU-004	2	NR	(*12*)
2014	Chile	NR	NR	NR	Septicemia	Recovered	NR	2	NR	(*13*)
2015	Chile	NR	NR	NR	Meningitis	Recovered	NR	2	NR	(*13*)
2018	Chile	44	F	Yes: Handling contact of 4 min	Meningitis	Hearing Loss	NR	2	NR	(*13*)
2019¶	Chile	NR	NR	NR	Meningitis	Recovered	P-194-2019	NR	1172	PubMLST
2011	French Guiana	42	M	Yes	Meningitis	Hearing Loss	NR	2	1	(*14*)
2008	Uruguay	66	M	Yes: Sausage factory worker	Meningitis	Hearing loss	NR	NR	NR	(*15*)
2009	Uruguay	53	M	Yes: Pig farmer	Meningitis	Recovered	NR	NR	NR	(*15*)
2009	Uruguay	NR	NR	NR	NR	Recovered	NR	NR	NR	(*15*)
2024	Uruguay	50	M	Yes: Raises pigs for	Meningitis	Recovered	NR	NR	NR	(*16*)

Year	Country	Patient characteristics					Isolate			Reference
		Age	Sex	Contact with swine	Disease manifestation	Outcome	Isolate ID	Serotype	ST	
				personal consumption						
2024	Uruguay	51	M	Yes: Raises pigs for personal consumption	Meningitis	Recovered	NR	NR	NR	(*16*)

*NR, not reported; ST, sequence type as determined by multilocus sequence typing; STSLS, streptococcal toxic shock–like syndrome.
†This infection is reclassified as *S. parasuis* in this study (please see main text).
‡This case was reported in 2024, but the actual infection timing was not provided.
§This patient was diagnosed with STSLS, but CSF findings were consistent with meningitis, suggesting concurrent central nervous system involvement despite an unremarkable CT scan.
¶This is a potential case based on a submission to PubMLST of a human *S. suis* isolate. However, it is unclear whether the isolate represent one novel infection or is associated with one of the previous seven infections in Chile.

References

1. Callejo R, Zheng H, Du P, Prieto M, Xu J, Zielinski G, et al. *Streptococcus suis* serotype 2 strains isolated in Argentina (South America) are different from those recovered in North America and present a higher risk for humans. JMM Case Rep. 2016;3:e005066. PubMed https://doi.org/10.1099/jmmcr.0.005066

2. Lopreto C, Lopardo HA, Bardi MC, Gottschalk M. Primary *Streptococcus suis* meningitis: first case in humans described in Latin America [in Spanish]. Enferm Infecc Microbiol Clin. 2005;23:110. PubMed https://doi.org/10.1157/13071618

3. Nagel A, Manias V, Busquets N, Sniadowsky S, Anzardi J, Méndez EL. *Streptococcus suis* meningitis in an immunocompetent patient [in Spanish]. Rev Argent Microbiol. 2008;40:158–60. PubMed

4. Núñez JM, Marcotullio M, Rojas A, Acuña L, Cáceres M, Mochi S. First case of meningitis by *Streptococcus suis* in the northwest area of Argentina [in Spanish]. Rev Chilena Infectol. 2013;30:554–6. PubMed https://doi.org/10.4067/S0716-10182013000500014

5. Callejo R, Prieto M, Salamone F, Auger JP, Goyette-Desjardins G, Gottschalk M. Atypical *Streptococcus suis* in man, Argentina, 2013. Emerg Infect Dis. 2014;20:500–2. PubMed https://doi.org/10.3201/eid2003.131148

6. Kerdsin A, Hatrongjit R, Wongsurawat T, Jenjaroenpun P, Zheng H, Chopjitt P, et al. Comparative genome analysis of *Streptococcus suis* serotype 5 strains from humans and pigs revealed pathogenic potential of virulent, antimicrobial resistance, and genetic relationship. Microbes Infect. 2025;27:105273. PubMed https://doi.org/10.1016/j.micinf.2023.105273

7. Santoya Espinosa LC, Morán Díaz DF, Díaz Aguiar P, Rodríguez Arias EA. *Streptococcus suis* meningitis [in Spanish]. Medicina (B Aires). 2024;84:329–32. PubMed

8. Ramos GSS, Rego RFDS, Oliveira MFF, Rocha VFD, Oliveira EP, Reis JN. *Streptococcus suis* meningitis: an emerging zoonotic disease in Brazil. Rev Soc Bras Med Trop. 2024;57:e00805. PubMed https://doi.org/10.1590/0037-8682-0610-2023

9. de Matos FBP, Farias LABG, Dias CN, Mendes LP, Bessa PPdN. Meningitis due to *Streptococcus suis* in two patients with occupational exposure from northeastern Brazil. Case Rep Infect Dis. 2021;2021:5512303. PubMed https://doi.org/0.1155/2021/5512303

10. da Silva NCZ, Varaschin PA, Castanho CR, dos Santos RS, Camargos VS, Garcia RAM, et al. *Streptococcus suis* meningitis in humans: the cause of emerging bacterial infectious diseases in Brazil? Case report. SN Compr Clin Med. 2020;2:2933–4. https://doi.org/10.1007/s42399-020-00617-1

11. Razera RJ, Santos-Oliveira JV, Boaventura-Santos M, Almeida-Pontes V, Kanegae MY, Ardengh JC. A rare cause of septicemia after pork meat ingestion. Cureus. 2024;16:e62096. PubMed https://doi.org/10.7759/cureus.62096

12. Alarcón LP, Araya RP, Aguayo C, Fernández J, Illesca V, Zaror A, et al. Laboratory confirmation of *Streptococcus suis* in Chile [in Spanish]. Rev Chilena Infectol. 2013;30:539–40. PubMed https://doi.org/10.4067/S0716-10182013000500011

13. Moraga J, Fica A, Navarrete B, Henríquez R, Navarrete-Asenjo C. Meningitis and hypoacusia due to swine meat handling [in Spanish]. Rev Chilena Infectol. 2018;35:731–2. PubMed https://doi.org/10.4067/S0716-10182018000600731

14. Demar M, Belzunce C, Simonnet C, Renaux A, Abboud P, Okandze A, et al. *Streptococcus suis* meningitis and bacteremia in man, French Guiana. Emerg Infect Dis. 2013;19:1545–6. PubMed https://doi.org/10.3201/eid1909.121872

15. Ventura V, Soca A, Noveri S, Seija V, Perendones M, Sartori G. *Streptococcus suis* meningoencephalitis: first two cases reported in Uruguay [in Spanish]. Arch Med Int (Montev). 2015;37:80–2.

16. Grasiuso L, Rodríguez F, Aguerrebere P, Bone G, Pan C, Alzugaray P. Acute meningoencephalitis due to *Streptococcus suis*. Case report [in Spanish]. Rev Med Urug (Montev). 2024;40:e701. https://doi.org/10.29193/RMU.40.3.7

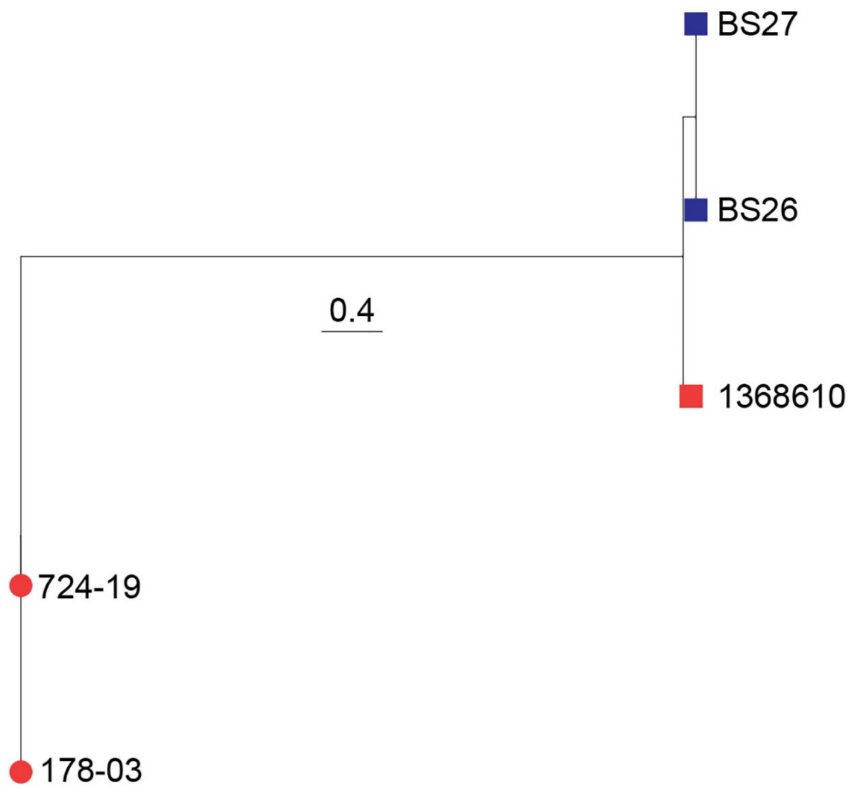

Appendix Figure. Phylogenetic relationships between *Streptococcus parasuis* human isolates from Argentina and People's Republic of China, and two representative ST1 serotype 2 *S. suis* isolates from Argentina. Depicted is a maximum-likelihood phylogenetic tree constructed using 5,504 non-redundant core-genome SNP loci identified among the isolates relative to the genome sequence of the *S. suis* ST1 serotype 2 reference strain P1/7 (not included in the depiction). The Argentine *S. parasuis* isolate 1368610 had previously been identified as *S. suis* serotype 21, but an ad hoc in silico PCR with specific primers targeting the *recN* genes of various streptococcal species reclassified the isolate as *S. parasuis* (see the main text). Supporting this reclassification, the phylogenetic analysis demonstrated that the isolate is genetically distant from other *S. suis* isolates but genetically close to Chinese *S. parasuis* isolates BS26 and BS27 (GenBank accession numbers CP069079.1 and JAETXU000000000.1, respectively).
